# Acromegaly among a Moroccan population

**DOI:** 10.11604/pamj.2023.46.116.41952

**Published:** 2023-12-27

**Authors:** Ahmed Anass Guerboub, Jad Issouani, Kesly Jeny Joumas, Yassine Er Rahali

**Affiliations:** 1 Endocrinology Department, Mohammed V Military Academic Hospital, Faculty of Medicine and Pharmacy, Mohammed V-Souissi University, Rabat, Morocco

**Keywords:** Acromegaly, growth hormone, dysmorphytic syndrome, pituitary adenoma

## Abstract

Acromegaly is defined as an acquired dysmorphytic syndrome due to excessive secretion of growth hormone (GH) and consequently of insulin-like growth factor-1 (IGF-1). This is a retrospective study of patients who were hospitalized in the Endocrinology Department of the Mohammed V Military Academic Hospital in Rabat over a period of 14 years (2008 to 2022), reporting on their clinical, paraclinical and evolutionary profiles and comparing the results with the data in the literature. Nineteen patients were included in our study. The mean age was 42.7 ± 11.6 years, with a male predominance. The clinical manifestations were dominated by a dysmorphic syndrome present in 97.4% of cases, followed by complications related to acromegaly in 88.9% of cases. The diagnosis was made when GH and IGF-1 values were elevated in 88.9% and 93.8% of cases, respectively; with a mean GH value of 25.1 μg/L. Magnetic resonance imaging (MRI) was used to diagnose the location of pituitary adenoma in all cases, 78.9% of which were macroadenomas and 21.1% microadenomas. The majority of patients (78.9%) had recourse to transsphenoidal surgery. Medical treatment was carried out in 89.5% of cases. Postoperative radiotherapy was performed in 33% of cases. Disease control was achieved in 30.1% of cases. This study shows the complex management of acromegaly. Disease control is a necessary condition in order to avoid complications, but is often difficult to obtain.

## Introduction

Acromegaly is a disorder characterized by a slowly progressive acquired dysmophic syndrome due to a persistent excess of growth hormone (GH) and consequently of insulin-like growth factor-1 (IGF1) [1,2]. While most cases are secondary to a GH-secreting pituitary adenoma [3], acromegaly can exceptionally be secondary to hypothalamic hypersecretion of growth hormone-releasing hormone (GHRH) or ectopic secretion of GH or GHRH [4]. The aim of our work is to trace the epidemiological, clinical, paraclinical, therapeutic, and evolutionary profile of acromegalic patients. We will also describe the recommended management of these patients.

## Methods

The present work consists of a retrospective study of patients suffering from acromegaly; hospitalized in the Endocrinology Department of the Mohammed V Military Academic Hospital in Rabat during the period from October 2008 to February 2022. The selection of the patients studied was done by a search on the computerized register available in the endocrinology department with the search word “acromegaly”. The results obtained are compared with data from the literature.

The patients included in this study all met the criteria for diagnosis of acromegaly, i.e, elevated GH and/or IGF-1. Patients with suspected acromegaly with normal GH levels without elevated IGF-1 were excluded from this study. As well as patients whose records could not be found or were incomplete.

Data were collected using a data collection form. For all patients included in the study, we recorded the different demographic, anthropometric, clinical, biological, and morphological characteristics related to acromegaly and its consequences. Statistical analysis was performed using Jamovi software. Quantitative statistics are expressed as mean ± standard deviation for Gaussian curves and as median and quartiles for non-Gaussian curves. Qualitative statistics are expressed as percentages.

## Results

During the study period, 19 patients were hospitalized for acromegaly and satisfied the inclusion criteria. The mean age of our patients was 42.7 years ± 11.6 years with extremes ranging from 19 to 65 years. Five women (26%) and 14 men (74%) were included in our study, i.e. a sex ratio of 2,8. As for the antecedents, they were dominated by diabetes, found in 52.6% of the cases, followed by hypertension (21.1%), neuropathies (10.5%), dyslipidemia, hyperparathyroidism, and cardiopathy, each of them found in 5.3% of our patients. In our group, 63.2% of acromegaly cases were revealed by a dysmorphic syndrome observed by the family or by the patient himself; 21.1% of the patients presented complications: visual disturbances (10.5%), headaches (5.3%), asthenia (5.3%), diabetes (5.3%), sleep apnea syndrome (5.3%). In addition, 26.3% of cases were discovered incidentally during a check-up (15.8%) or during hospitalization (10.5%) (Table 1).

**Table 1:** different circumstances of diagnosis of acromegaly in our study (N = 19)

Revelation mode	Circumstances		Symptoms	Number of patients	Percentage
Entourage/patient	Dysmorphic syndrome		Thickened lips	12	63.2
			Prognathism		
			Curved forehead		
			Enlarged nose		
			Thickened hands and feet		
Incidental	Check-up		Routine check-up	3	15.8
	Hospitalization		Narrow lumbar canal surgery	1	5.3
			Herniated disc operation	1	5.3
Complications	Related to the tumoral process	Endocranial syndrome	Visual disorders	2	10.5
			Headaches	1	5.3
		Endocrine syndrome	Asthenia	1	5.3
		
	Related to GH hypersecretion		Diabetes mellitus	1	5.3
			OSAS	1	5.3

OSAS: obstructive sleep apnea syndrome; GH: growth hormone

The clinical manifestations were dominated by symptoms related to GH hypersecretion. The dysmorphic syndrome was present in 18 of our patients, i.e. 94.7% of the cases: a thickened nose in 18 patients (100%), prominent arches in 18 patients (100%), thickened lips in 16 patients (88.9%), hand and foot enlargement in 16 patients (88.9%), prognathism in 12 patients (66.7%), cheekbone protrusion in 10 patients (55.6%), interdental spacing in 4 patients (22.2%), spinal bone deformities in 13 patients (kyphosis (30.8%) and hyperlordosis (23.1%)), and head enlargement in 2 patients (11.1%). Follow-up with complications related to acromegaly present in 16 of our patients, i.e. 88.9%, represented by: diabetes (70.6%), Sleep Apnea Syndrome (SAOS) (75%), cardiopathy (mainly left ventricular hypertrophy) (55.6%), neuropathies (33.3%): mainly paresthesias (22.2%) or carpal tunnel syndrome (5.6%), arthropathies (40%), skin changes: hyperhidrosis (26.7%), seborrhea (20%), hypertension (15.8%). The splanchnomegaly was present in 16 patients, i.e. 84.2% of the cases, with: mainly macroglossia (77.8%), goitre (52.6%), hepatomegaly (11.1%), splenomegaly (5.6%), cardiomegaly (5.6%). The tumor syndrome was present in 11 patients, i.e. 61.1% of the cases, and was dominated by: visual disorders (27.8%), asthenia (27.8%), and headaches (22.2%). Finally, signs of hypopituitarism such as asthenia (27.8%), decreased libido (22.2%), depilation (11.1%), arterial hypotension, depigmentation, amenorrhea, and weight gain, each found in 1 case (5.6%). Biologically, all patients had a GH/IGF-1 test. The HGPO test was performed in 1 patient (but not found). Growth hormone (GH) was elevated in 88.9% of cases; IGF-1 was elevated in 93.8% of cases.

Hypothalamic-pituitary magnetic resonance imaging (H-P MRI), performed in all of our patients, revealed the presence of pituitary adenoma in all 19 patients, i.e. 100% of cases. In 15 of our patients (78.9% of the cases), presented a macroadenoma; while the other 4 (21.1% of the cases) showed a microadenoma. Pituitary adenomas can lead to compression of the optic chiasm, causing various visual disorders. Therefore, the evaluation of the endocranial impact of our patients was based on the ocular fundus, visual field examination, and visual acuity. Fundus and visual acuity were performed in 18 patients, while visual field examination was performed in 17 patients. Fundus examination was pathological in 6 patients (33.3% of cases), showing chorioretinal atrophy in 3 patients (16.7% of cases), diabetic retinopathy in 3 patients (16.7% of cases), subcapsular cataract in 2 patients (11.1% of cases). Five patients had a decrease in visual acuity (27.8% of cases). The visual field was affected in 8 patients, (56.3% of cases), with: a hemianopia found in 6 patients (37.5% of cases), a thickening of the blind spot in 2 patients (12.5% of cases), a quadrianopia found in 1 patient (6.3% of the cases).

The evaluation of the endocrine impact was based on the exploration of the different hypothalamic-pituitary axes. The mean cortisol level was 104 ± 43.4 ng/ml with a low level (26.5 ng/ml) found in 1 patient (5.9% of cases), while in the 16 others (94.1% of cases) the cortisol level was normal. Prolactin assay performed in 16 patients showed hyperprolactinemia in 3 patients (31.2% of cases). Thyroid insufficiency was present in 6 patients (35.3% of cases). Gonadotropic insufficiency was found in 10 patients (58.8% of cases). The metabolic impact in our study was assessed by means of blood sugar, phosphocalcic, lipid, and hepatic tests. Diabetes was found in 12 patients (70.6% of cases). Concerning the phosphocalcic balance, hyperphosphatemia was found in 2 patients (13.3% of cases); the calcemia and phosphoremia were normal in the other cases. Dyslipidemia was found in 8 patients (47.1% of cases). Elevated transaminases (aspartate aminotransférases/alanine aminotransferase (ASAT/ALAT)) were present in 4 patients (25% of cases). The respiratory impact assessment was based on polysomnography, performed in 12 patients. It showed an obstructive sleep apnea syndrome (OSAS) in 9 patients (75% of cases).

The evaluation of the cardiovascular complications in our patients was based on the electrocardiogram (ECG), transthoracic echocardiography (TTE) blood pressure monitoring (BP), and standard chest radiography. The ECG and TTE performed on 18 patients revealed the presence of left ventricular hypertrophy (LVH) in 6 patients (33.3% of cases), arrhythmias in 4 patients (22.2% of cases), valvulopathy in 2 patients (11.1% of cases), ischemic heart disease in 2 patients, and arterial hypertension (AH) in 3 patients (15.8% of cases). Organomegaly was sought in our study, mainly by cervical and abdominal ultrasound performed respectively in 15 and 16 patients. Were found: goiter in 10 patients (66.7% of cases), hepatomegaly in 6 patients (40% of cases), hepatosplenomegaly in 2 patients (12.5%), vesicular lithiasis and renal cysts in 3 patients (18.8% of cases).

Therapeutically, 15 patients (78.9% of the cases) had surgical treatment, all by trans-sphenoidal approach. No operative complications were recorded in 73.3% of the cases. 26.7% of the cases had some complications such as diabetes insipidus, hypopituitarism or visual impairment. Four patients had to undergo a second or third surgery. In addition to the patients who did not receive surgical treatment, the majority received medical treatment postoperatively, i.e. a total of 89.5% of cases were treated with somatostatin analogues (SSA) (lanreotide LP 120 mg at 1 injection/month). However, they were associated with bromocriptine in 1 patient, and with cabergoline in 7 patients (36.8% of cases). Postoperative radiotherapy was performed in 6 patients (33% of cases), with 2 patients receiving GammaKnife. The immunohistochemical study performed in 11 patients revealed the presence of: adenomas expressing only GH in 36.4% of cases. The remaining cases showed mixed adenomas expressing GH and prolactin in 36.4% of cases, GH and LH in 18.2%, GH, prolactin, and LH in 9.1% of cases, GH, LH, and FSH in 9.1% of cases.

Regarding the evolution; the initial evaluation was performed three months after surgery, with an IGF-1 dosage, a metabolic and endocrine assessment, and a control MRI. In 17 patients evaluated postoperatively, 8 (47.1% of the cases) had a favorable evolution without complications. Acromegaly was controlled in 26.7% of cases, and not controlled in the others (73.3% of cases). The duration of follow-up of the patients included ranged from 6 months to 15 years. Clinically, 14 out of 16 patients (87.5% of the cases) presented a persistence of their dysmorphic syndrome while an improvement was noted in the 2 others (12.5% of the cases). Biologically, the control IGF-1 was decreasing in 66.7% of cases while it was still increasing in 33.3% of cases. On the follow-up MRI, recurrence was noted in 7.7% of the cases; an increase in the tumor process was present in 7.7% of the cases and a stationary state in 7.7% of the cases. A reduction of tumoral volume was noted in 61.5% of cases.

## Discussion

In our study, the mean age at diagnosis was 42.7 years, in agreement with the data of other series, in particular, the study published by Pedro de Pablos-Velasco *et al*. [5] with a mean age of 47.6 ± 8.6 years. Concerning the sex ratio, our study reported 14 men/5 women, i.e. a male predominance, contrary to the results found in the literature which mark a female predominance [6,7]. However, in our series, the women were older (mean age = 48.8 years) than the men (mean age = 40.5 years) at the time of diagnosis [8], most of the female patients being postmenopausal (60% of the cases), as observed in all studies of acromegaly [8].

Acromegaly can be revealed accidentally during a biological examination, the observation of a dysmorphic syndrome by the physician, the patient himself or his entourage, or in front of complications of the disease. In our study, 63.2% of the patients consulted following the observation of a dysmorphic syndrome. As in our study, acromegaly is frequently suspected in front of a dysmorphic syndrome. However, in most of the series, this finding is made by the treating physician, more rarely by the family or the patient himself [9].

The diagnosis of acromegaly is delayed by a variable number of years due to its indolent and insidious nature. The duration between the onset of symptoms and the diagnosis of acromegaly varies from 6.6 to 10.2 years, with an average delay of about 9 years. This latency period to diagnosis seems to have shortened, probably reflecting improved physician awareness, the availability of more sensitive diagnostic tools and the increased use of MRI [10]. In our study, out of 7 patients, the diagnostic delay was indeed shorter, ranging between 1 and 8 years with a mean delay of 3.29 years.

The diagnosis of acromegaly is made when clinical signs are present (Table 2), with an elevated age-adjusted IGF-1 concentration and a lack of suppression of GH levels (GH<1 μg/L) after administration of an oral glucose load. Most patients also have an elevated basal GH concentration [11].

**Table 2:** comparison of the frequency of symptoms and complications found at the time of diagnosis between our group and the literature

Syndromes and complications	Symptoms	Patients (%)			
		Our study	AlMalki *et al*.	Caron *et al*.	Carosi *et al*.
Dysmorphic syndrome	Enlargement of the extremities	88.9	90	89	100
	Changes in facial features	100	89.8	83	-
	Prognathism	66.7	85.5	45	-
	Dental issues	22.2	75	35	-
Tumoral syndrome	Headache	22.2	72.8	62	28
	Visual disorders	27.8	51.6	28	-
	Asthenia	27.8	-	75	-
	Decreased libido	22.2	52.4	45	-
	Weight gain	5.6	68.8	65	-
	Amenorrhea	5.6	31.2	63	-
Splanchnomegaly	Macroglossia	77.8	67.6	45	-
	Goiter	52.6	37.5	-	52
	Cardiomégaly	5.6	-	15	-
Complications	Diabetes mellitus	70.6	60.7	28	36
	Arterial hypertension	15.8	50	40	60
	Cardiopathy	55.6	20.2	5	36
	OSAS	75	47.8	50	-
	**Neuropathy**				
	Paresthesia	22.2	-	-	40
	carpal tunnel syndrome	5.6	31.5	58	36
	Hyperhidrosis	26.7	70.8	70	12
	Skin thickening	26.7	-	45	-
	Arthropathy	40	35.4	68	40

OSAS: obstructive sleep apnea syndrome

The mean GH value at diagnosis was 51.17 mIU/L (17 μg/L) and 57.19 mIU/L (19.04 μg/L), respectively for men and women [9]. In our study, the mean GH value was 25.1 μg/L, with extremes ranging from 0.4 μg/L to 220 μg/L. In the study by Carosi *et al*. [9], IGF-1 was elevated in 85.6% of cases. These results are similar to our study, in which IGF-1 was elevated in 93.8% of cases. In the series by AlMalki *et al*. [10], elevated serum GH levels not suppressible below 1 ng/dL after an oral glucose load of 75 g were observed in 30 patients (15.4%) of the total cohort. In our series, only one *L'hyperglycémie provoquée par voie orale* (HGPO) test was performed, but the results were missing.

Hypothalamic-pituitary magnetic resonance imaging (H-H MRI) is the reference examination for localization in acromegaly. In our series, 100% of the patients underwent hypothalamic-pituitary MRI. it revealed a macroadenoma in the majority of cases (78.9% of cases) while the others (21.1% of cases) presented a pituitary microadenoma. These results correspond to those of Caron *et al*. [6], who show a predominance of macroadenomas (80.5% of cases) compared to microadenomas (19.5% of cases). Concerning the impact of acromegaly, in our study, visual field damage was predominant, present in 56.3% of cases, higher than in the study of AlMalki *et al*. [10] where it was present in 30.5% of cases. Hypopituitarism is caused by compression or damage to the pituitary gland by adenoma expansion. It can also be an iatrogenic complication after surgery or radiotherapy. The prevalence of hypopituitarism in the study of Rolla *et al*. [12], was 37% and it was dominant in the male population. In our research, it was present in 58.8% of cases.

Growth hormone (GH) is an insulin antagonist hormone. It increases lipolysis, and gluconeogenesis and predisposes to insulin resistance [12]. The prevalence of diabetes in patients with acromegaly varies from 12 to 53% [12-14], in comparison to our study where it was higher (70.6% of cases). In the study of Warncke *et al*. [15], it has been reported that diabetes in acromegaly affects younger patients (50.1 years) than in the general population (59 years). This finding is also valid in our study with an average age of 45.4 years.

Regarding dyslipidemia, depending on the source, it affects 13-51% or even up to 71% of patients with acromegaly [15,16]. Hypertriglyceridemia and decreased high-density lipoprotein (HDL) levels are the main lipid abnormalities in acromegaly [16]. In the series of Rolla *et al*. [12], dyslipidemia was the most frequent complication in the whole group (observed in 74% of cases), its incidence being higher compared to previous studies [15] as well as in ours, where it was found in 47.1% of cases.

In patients with acromegaly, an upper airway obstruction may develop. It is due to the enlargement of the tongue and the thickening of the laryngeal tissues. Thus, acromegaly is often associated with obstructive sleep apnea syndrome (OSAS). Although the obstructive apnea type has been considered predominant, some reports suggest that central apnea episodes have a high rate. They are related to abnormalities of central respiratory control. The presence and extent of central sleep apnea in acromegaly are unresolved issues. The morphological changes so characteristic of acromegaly determine obstructive apnea. However, the hormonal milieu that determines the predominance of apnea type is not yet well understood [17]. In the study of Chaouki *et al*. [18], OSAS was present in 76.2% of cases, corresponding perfectly to our study results with a prevalence of 75% of cases.

Cardiovascular complications are the major causes of mortality in patients with acromegaly [16,19]. Cure or even control of the disease activity, decrease the frequency and severity of comorbidities in this group. Some of them may even be completely reversed [20]. Arterial hypertension was the dominant cardiovascular complication in the series of Rolla *et al*. [12] with a prevalence of 58%, followed by valvulopathies and ventricular hypertrophy in 34% of cases, arrhythmias (7 - 48%) and ischemic heart disease (7% of cases). In our study, it was left ventricular hypertrophy (LVH) that predominated (in 33.3% of cases), followed by arrhythmias (22.2%). Arterial hypertension was present in 15.8% of cases. In the study of Rolla *et al*. [12], densitometry was performed in 47.4% of cases, revealing osteoporosis in 12 - 32% of cases, which is similar to our study (28.6% of cases). This decrease in bone density, common in acromegaly, is regularly associated with an increased prevalence and incidence of vertebral fractures. However, bone density may be normal on standard Dual X-ray absorptiometry (DXA) as in other forms of endocrine osteopathy´s [21].

Acromegaly is associated with a 20% increase in large bowel length and an increased prevalence (37% of patients) of colonic diverticula that correlate with GH and IGF-1 levels at diagnosis [22]. Patients with active acromegaly have increased proliferation of colonic epithelial cells that is associated with elevated IGF-1 levels. Therefore, colonic polyps are the most common tumors in acromegaly requiring screening by colonoscopy due to the risk of malignant transformation. The prevalence of colonic polyps and adenomas is related to the duration of uncontrolled acromegaly. The risk of colon cancer is higher in younger patients with acromegaly, while IGF-1 levels or disease duration are not associated with the prevalence of colonic neoplasia [22]. In the series of AlMalki *et al*. [10] and Caron *et al*. [6], colonic polyps were present in 22.7% and 27% of cases respectively, consistent with our study, in which they were found in 25% of cases.

Regarding organomegaly, the most common symptom is a goiter. In the literature, the rate of goiter in this population reaches 92%. Indeed, several studies have shown a positive correlation between the volume of the thyroid gland and the levels of GH and IGF-1. In addition, a decrease in thyroid volume has been noted one year after initiation of octreotide therapy, or after successful surgery [22]. Nodular goiter has been reported in 39 to 65% of patients with acromegaly with a correlation to the duration of the disease. The rate of thyroid cancer varies in different reports between 1.2% and 10.6%. In our series, goitre was the majority and was found in 66.7% of cases. However, no cancer was found.

Surgery is generally considered the primary treatment, with initial remission rates of 85% for microadenomas and 40% to 50% for macroadenomas. However, 40% to 60% of patients will experience recurrent or persistent disease after surgery, requiring additional treatment [10]. Medical treatment or radiotherapy is usually reserved for a tumor that cannot be completely resected by surgery, or contraindication to surgery, also in case of uncontrolled disease. In the study by AlMalki *et al*. [10], 95% of the patients underwent surgery, mostly by transsphenoidal approach (98%); only 3 patients required transcranial approach, while in 10.3% of the cases, no surgical data was available. In our series, 15 patients, i.e. 78.9% of the cases, had recourse to surgical treatment and exclusively by transsphenoidal approach (100%).

In the study by AlMalki *et al*. medical treatment was used for the majority of patients mainly as an adjunct to surgery. Of these, 74.4% (122/164) had been treated with long-acting somatostatin analogues (SA), mainly octreotide LAR. Only 3 patients were treated with pasireotide after failure of octreotide LAR. While dopamine agonists (bromocriptine or cabergoline) were used in 3.2% (3/95) and 32% (44/139), respectively. In addition, when data were available for 124 patients, 8% were treated with the GH receptor antagonist pegvisomant [10]. In our study, 89.5% of patients were treated with SA, mainly lanreotide autogel LP 120 mg at 1 injection/month. Subsequently, it was combined with bromocriptine in 1 patient (5.3%), and mainly with cabergoline in 7 patients (36.8%). None of our patients was treated with pegvisomant.

The data of AlMalki *et al*. [10] showed that radiotherapy was used in 24% of the patients (42/174), mainly as adjuvant treatment after surgery. In our study, postoperative radiotherapy was performed in 33% of patients also as an adjunct to surgery. Anatomopathological examination with immunohistochemical study of the pituitary gland removed during surgery is systematic. The purpose is to ensure the tissuular nature of lesions, to specify the benign or malignant character and to determine the type of adenoma. In the literature, pure somatotropic adenomas are the most frequent, followed by impure somatotropic adenomas with GH and prolactin (PRL); while the other forms are found less frequently [23]. In our series, pure somatotropic adenomas (36.4%) and impure somatotropic GH and prolactin adenomas (36.4%) were the most frequent forms. As for the other forms, they were higher in our series (27.2% of cases) than in the other studies (3% of cases).

The evolution and prognosis of acromegaly depend on the management and control of this disease. Uncontrolled acromegaly is a well-established cause of increased morbidity and mortality with an overall standardized mortality ratio (SMR) approximately 2 times that of the baseline population [24]. The incidence of postoperative complications for pituitary adenomas is highly variable. The choice of surgical visualization (microscopic versus endoscopic), the extent of the surgical approach (standard versus transsphenoidal), the extension of the sellar versus parasellar tumor, the heterogeneity of the reported series, and the experience of the surgical team are factors that may account for this variation [25]. In our series, 26.7% of the operative sequelae were complicated by diabetes insipidus, hypopituitarism or ocular injury mainly. In the series by Mortini *et al*. [26], hypopituitarism was also found, but at a lower frequency with hypogonadism in 1.5% of cases, hypothyroidism in 0.5% and hypoadrenalism in 1.1% of cases.

In the short term, 41.1% of our patients had a favorable evolution without complications. The control of acromegaly (GH < 2.5 μg/l associated with normalization of IGF-1) postoperatively and associated with medical treatment, was achieved in 26.7% of cases. In the series of AlMalki *et al*. [10], the cure rate with multimodal treatment was 28.7%, which is very similar to our study results. Moreover, 30.1% of patients were not cured by surgery and radiotherapy, but good control of symptoms and biochemical markers was achieved with medical treatment, while 28.2% of patients still had active disease despite the use of multiple treatment modalities.

Long-term biochemical control is achieved in less than 65% of patients after surgical resection, and only half of patients treated with medical therapy achieve control of IGF-1 levels. Radiotherapy remains an option in patients with persistent active disease, but control and safety rates have improved only slightly with the use of stereotactic radiosurgery instead of conventional fractionated radiotherapy. The management of acromegaly and its associated comorbidities is complex and requires a comprehensive approach coordinated by a multidisciplinary team of expert in the management of pituitary tumors [27]. In our study, clinically, 87.5% of the cases presented a persistence of their dysmorphic syndrome. Biologically, the control IGF-1 was decreasing in 66.7% of the cases while it was increasing in 33.3% of the cases. On the follow-up MRI, a reduction of tumoral volume was noted in 61.5% of cases.

## Conclusion

Acromegaly is a rare disease. It evolves spontaneously, towards serious and sometimes fatal complications. A methodical management allows to optimize the control and to reduce the excess mortality related to this pathology.

### What is known about this topic

- *Acromegaly is a disorder characterized by a slowly progressive acquired dysmophic syndrome due to a persistent excess of growth hormone (GH) and consequently of insulin-like growth factor-1 (IGF1);*
- *The evolution and prognosis of acromegaly depend on the management and control of this disease;*
- *Uncontrolled acromegaly is a well-established cause of increased morbidity and mortality*.

### What this study adds

- *This work outlines the epidemiological, clinical, and paraclinical particularities of acromegaly in the Moroccan context;*
- *Assessing the efficiency of different therapeutic approaches;*
- *Evaluation of management impact on long-term outcome of acromegalic patients*.
